# Anatomical Study of the Cervical and Interosseous Talocalcaneal Ligaments of the Foot with Surgical Relevance

**DOI:** 10.7759/cureus.1382

**Published:** 2017-06-22

**Authors:** Alisha J Poonja, Mika Hirano, Djavlon Khakimov, Naomi Ojumah, R. Shane Tubbs, Marios Loukas, Piotr B Kozlowski, Khurram H Khan, Anthony C DiLandro, Anthony V D'Antoni

**Affiliations:** 1 Department of Pre Clinical Sciences, NYCPM; 2 SGU Department of Anatomical Sciences, Seattle Science Foundation; 3 Neurosurgery, Seattle Science Foundation; 4 Department of Anatomical Sciences, St. George's University School of Medicine, Grenada, West Indies; 5 Neurology, Neuromedlab; 6 Department of Podiatric Medicine, Temple University, School of Podiatric Medicine; 7 Division of Pre Clinical Sciences, New York College of Podiatric Medicine; 8 Department of Molecular, Cellular and Biomedical Sciences, CUNY School of Medicine

**Keywords:** anatomy, stability, clinical, intervention, morphology, injury, surgery, podiatry

## Abstract

There is conflicting evidence regarding the morphology and orientation of the cervical ligament (CL) and interosseous talocalcaneal ligament (ITCL). The morphology of the CL and its relationship to the ITCL were studied to obtain an understanding of these structures. Twenty-six feet (13 left, 13 right) were obtained from formalin-fixed cadavers (14 females, four males) with the mean standard deviation (SD) age at death 80.9 (12.9) years. All measurements were made with a digital caliper. The length and width of the foot, the width and height of the talus, were measured. The talus was cut coronally to expose the ITCL and qualitative observations were noted. The mean (SD) heights and widths of the CL at the anterior, posterior, superior, and inferior points were 8.27 (2.52), 13.95 (5.96), 9.15 (2.45), and 11.90 (4.30) mm, respectively. The mean (SD) thicknesses of the CL at the superoanterior, superoposterior, inferoanterior, inferoposterior, and central points were 0.62 (0.24), 1.05 (0.30), 0.70 (0.26), 1.20 (0.34), and 0.97 (0.31) mm, respectively. The fibers of the CL are oriented at a slight superoanterior to inferoposterior angle, whereas the fibers of the ITCL are oriented in a slight superomedial to inferolateral angle. The fibers of the CL and ITCL overlap inside the tarsal sinus with the CL positioned anteriorly, which helps to distinguish the two ligaments. In this study, we identified the morphometrics of the CL and described the CL and ITCL qualitatively. These results are relevant to introducing innovative techniques for reconstructive surgery of the subtalar ligaments in order to repair, for example, subtalar instability.

## Introduction

Inversion ankle sprains are common injuries and it is estimated that 23,000 ankle sprains occur per day in the United States, for which approximately 55% of patients do not seek treatment [1]. Lateral ankle sprains typically occur when the rear foot is supinated and the leg externally rotated [1]. The most common predisposing factor for a lateral ankle sprain is a history of at least one previous ankle sprain, and individuals who suffer numerous sprains have been reported as having functional, chronic, or residual ankle instability [1]. Injuries to the lateral ankle and subtalar ligaments can occur concomitantly from inversion sprains, and the ligaments most commonly involved in sprains are the lateral ankle ligaments (anterior talofibular, calcaneofibular, and posterior talofibular) and the subtalar ligaments (cervical and interosseous talocalcaneal). Tears of the lateral ligaments are considered as pathophysiological contributors to subtalar instability, which results from the loosening of the subtalar ligaments, specifically the interosseous talocalcaneal and cervical ligaments.

The main stabilizing ligaments of the subtalar joint (STJ) are the cervical ligament (CL) and the interosseous talocalcaneal ligament (ITCL). The primary function of the CL is to resist excessive STJ supination whereas the ITCL remains taut during pronation. Dysfunction of either the CL or ITCL can result in subtalar instability, resulting in a variety of biomechanical pathologies presenting not only in the foot and ankle but also extending proximally to the knees, hips, and lower back. The ITCL has been described as running just posterior to the CL and the two ligaments are said to cross within the sinus and canalis tarsi [1]. The ITCL and CL are described as the “cruciate ligaments of the subtalar joint” [1]. However, the exact morphology of the CL and ITCL and their precise relationship within the canalis tarsi are poorly understood.

The CL and ITCL are often overlooked during diagnosis and/or treatment of lateral ankle sprains. Currently, subtalar fusion is the preferred treatment for painful subtalar instability. However, reconstructive ligament repair can also be considered for patients who do not tolerate or consent subtalar fusion. Many studies have examined the ligamentous ITCL [2-4]. An ITCL reconstruction was performed using a partial Achilles graft [2], whereas, in another study [3], the anterior portion of the peroneus brevis was used. Most recently, a successful reconstructive ligament repair of the ITCL using a gracilis autograft under subtalar arthroscopy was reported in an 18-year-old male following an ankle inversion injury [4]. It was reported that the patient’s clinical symptoms were successfully reduced [4]. Patients in all three studies demonstrated positive results, revealing ligamentous reconstruction as a possible treatment for subtalar instability meriting further investigation. The precise dimensions of and relationship between the subtalar ligaments would be beneficial for such reconstructive subtalar ligament procedures.

The precise dimensions and relationship of the subtalar ligaments could also be used in the prevention and diagnosis of sinus tarsi syndrome. Sinus tarsi syndrome is characterized by persistent anterolateral ankle pain, usually as a result of traumatic injuries to the ankle. The subtalar ligaments are frequently torn in individuals with sinus tarsi syndrome. An arthroscopic study was able to demonstrate defects in both the cervical and interosseous talocacalneal ligaments [5]. The CL in patients with sinus tarsi syndrome contained a partial tear in 33% of patients, arthrofibrosis in 24%, and soft tissue impingement in 21%. Similarly, the ITCL had a partial tear in 86% of ligaments or synovitis in 55% of patients [5]. Understanding the variations in size and morphometrics of the CL and ITCL could help predict the degree of motion of the subtalar joint and possibly the severity of pain in patients suffering from sinus tarsi syndrome.

There are significant discrepancies in the literature regarding the nomenclature, morphology, and function of the subtalar ligamentous complex, especially the structures most commonly referred to as the CL and ITCL [1]. 

The purpose of this study was to examine the precise anatomical relationships between the CL an ITCL and to discuss possible clinical correlations related to the primary ligamentous stabilizers of the subtalar joint. The authors consider the CL and ITCL to be distinct ligamentous structures that overlap within the sinus tarsi; however, we are interested in examining their specific relationship. Anatomical sharp and blunt dissection was used to expose the CL and its length and thickness were measured at various points using a digital caliper (Hawk Inc., Cleveland, Ohio). A Stryker saw (Stryker Corporation, Michigan, United States) was used to cut the talus in the coronal plane to expose the ITCL, its length and thickness were also measured at various points using a digital caliper. The dimensions and precise relationship between the two subtalar ligaments were examined and described.

## Materials and methods

A total of 13 right and 13 left feet were dissected. The feet were obtained from 18 formalin-fixed cadavers comprised of 14 females and four males with an average age at death of 80.9 years. Table 1 summarizes the demographics of the sample in this study. The lower limbs were cut about 10 cm above the medial and lateral malleoli with a Stryker saw in order to maneuver the feet easily.

**Table 1:** Demographics representation of sample (N=18)

Characteristics - Age group (years)	Cadaver No. (%)
51 - 60	2 (11.1)
61 - 70	1 (5.6)
71 - 80	4 (22.2)
81 - 90	8 (44.4)
91 - 100	3 (16.7)
Sex	
Female	14 (77.8)
Male	4 (22.2)

**Figure 1:**
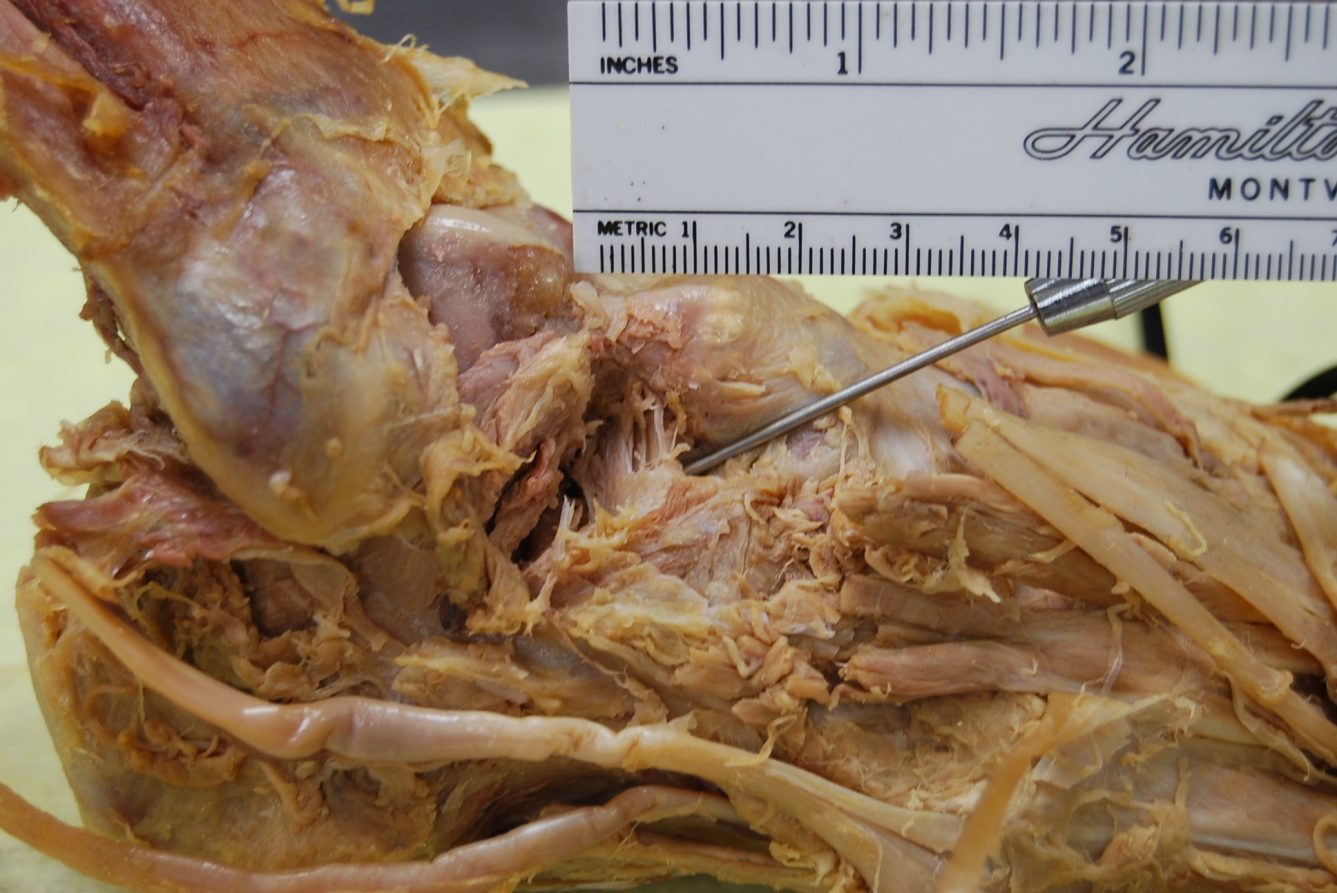
Figure showing right foot with over lying soft tissues dissected away to illustrate the cervical ligament (over pointer)

**Figure 2:**
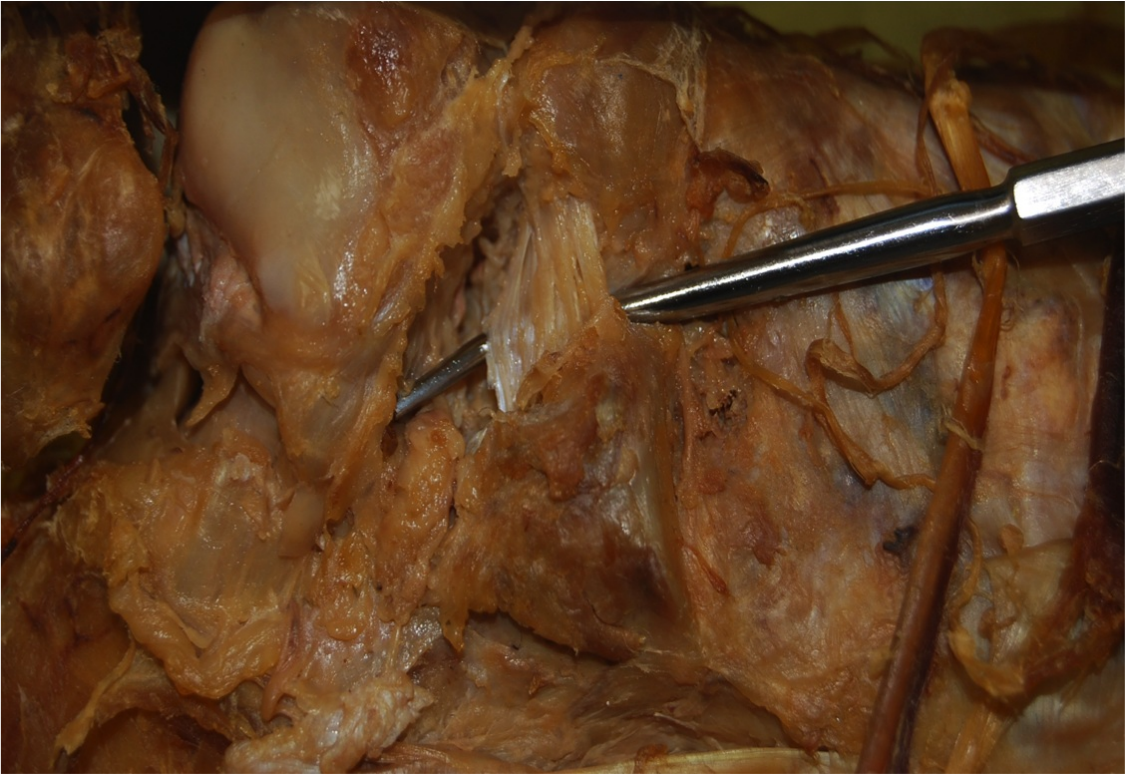
Figure shown as an enlargement of figure 1

**Figure 3:**
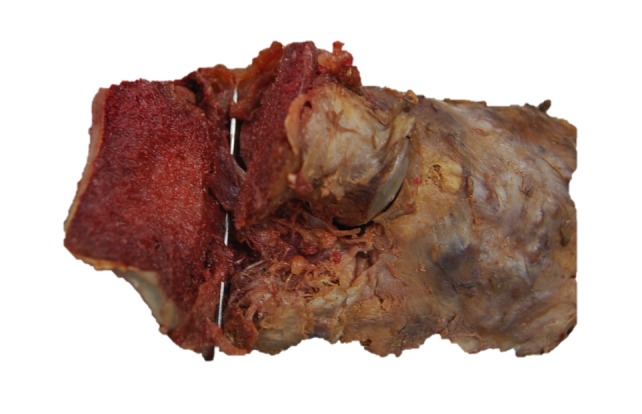
Figure showing the dissection of the talocalcaneal interosseous ligament (over pointer) following axial section through the talus

Superficial incisions were made to dissect the skin and deep fascia off completely. The superior and inferior extensor retinacula were cut to expose the tendons. The tendons of the extensor digitorum longus, tibialis anterior, extensor hallucis longus, fibularis longus, and fibularis brevis were cut at their insertions. The muscle belly of the extensor digitorum brevis and its tendons were cut to expose the underlying bones and ligaments. The length of the foot was measured using a ruler from the most posterior end of the calcaneus to the most anterior end of the second distal phalanx. Its width was measured with a ruler at the level of the metatarsal heads. The subtalar joint was cleaned with a probe to expose the CL clearly. The width and height of the cervical ligament (Figure 1-2) were measured. Its thickness was measured at the superior anterior, superior posterior, inferior anterior, inferior posterior and center points with a digital caliper (Hawk Inc., Cleveland, Ohio). The width and height of the talar neck were measured using a digital caliper (Hawk Inc., Cleveland, Ohio). The body of the talus was cut in the frontal plane and its proximal portion was removed to expose the ITCL (Figure 3). Descriptions of the ITCL were recorded for each cadaver.

The measurements were statistically analyzed using SPSS Version 20.0 (IBM, Armonk, New York) to find the mean values and to determine any correlations among them. A p-value of 0.05 was used as the criterion of statistical significance.

## Results

The average foot lengths and widths were 21.71 and 7.84 cm, respectively. The average talar neck height and width were 15.01 and 25.92 mm, respectively. The average height of the CL was 8.27 mm at the anterior end and 13.95 mm at the posterior. The average width of the cervical ligament was 9.15 mm at the superior end and 11.90 mm at the inferior. The average thickness of the CL was 0.62 mm at the anterosuperior aspect, 1.05 mm at the posterosuperior aspect, 0.70 mm at the anteroinferior aspect, 1.20 mm at the posteroinferior aspect, and 0.97 mm at the center of the ligament. Table 2 summarizes the morphometric data in this study.

**Table 2:** Morphometrics Data ^a^ = measured from head to second distal phalanx to calcaneus ^b ^= measured from medial aspect of the 1st metatarsal to the lateral aspect of the fifth metatarsal head

Cervical ligament	Mean (Standard Deviation-SD)
	Height
Anterior point	8.27 (2.52)
Posterior point	13.95 (5.96)
	Width
Superior Point	9.15 (2.45)
	Thickness
Superoanterior edge	0.62 (0.24)
Superoposterior edge	1.05 (0.30)
Inferoanterior edge	0.70 (0.26)
Inferoposterior edge	1.20 (0.34)
	Height
Talar neck	15.01 (2.61)
	Width
Talar neck	25.92 (2.17)
	Lentgtha
Foot	21.7 (1.71)
	Widthb
Foot	7.84 (0.77)

The CL appears to be a bundle of fibers and has a striated appearance. It increases in size posteriorly and superiorly, as demonstrated by the average height and width. One hundred percent of the CLs were oriented in a nearly vertical fashion but had a slight angle superoanteriorly to posteroinferiorly, some more than others. There were a few significant correlations among the variables measured in this study. Table 3 summarizes the correlations, the Pearson r values and the p-values.

**Table 3:** Correlations of data

p-value	Pearson r	N	Correlated variable
CL			
0.011	-0.498	25	Height (Posterior) x Thickness (Superoanterior edge)
0.040	0.406	26	Width (Inferior) x Height (Anterior)
0.019	-0.464	25	Width (Inferior) x Thickness (Superoanterior edge)
0.038	-0.425	24	Width (Superior) x Thickness (Inferoanterior edge)
Other			
0.012	0.477	27	Foot Length x Talar Neck Height
0.015	0.481	25	Talar Neck Height x CL Width
0.027	0.920	5	Talar Neck Width x CL Thickness (Infeorposterior edge)

The ITCL (Figure 3) was observed as a bundle of fibers that spanned across the entire width of the sinus tarsi. Its striated bundle of fibers was oriented in a superomedial to inferolateral fashion. The angles of the ITCL were greater in some cases than others. The ITCL overlaps with the CL in the sinus tarsi, but the distinction between the two ligaments is apparent from the orientations of their fibers. The CL was also situated anteriorly with respect to the ITCL in all of the cadavers studied.

## Discussion

The CL and ITCL are deep and important stabilizing ligaments of the subtalar joint [1]. The CL runs diagonally at about a 45° angle through the tarsal sinus with the proximal attachment to the superolateral surface of the calcaneus and the distal attachment to the talar neck [4, 6]. CLs were found to contain two or more bands [6]. In our study, the CL was consistently found to contain a single thick band with multiple fibers aligned in parallel. Furthermore, although most specimens showed the oblique orientation of the CL, a few had a vertically oriented cervical ligament with a slight angle suggesting various adaptations of this ligament in response to stresses associated with various activities. Some anatomy atlases identify the CL as the ITCL without distinguishing the two [7]. However, while they are continuous deep inside the tarsal sinus, the CL is positioned anteriorly coursing obliquely in a superior direction in most specimens, as seen in our study. In a particular study [3], it is suggested that sectioning the CL increases the angle between the talus and the calcaneus in the frontal plane when the foot is supinated and the leg is externally rotated. In other words, the CL resists supination at the subtalar joint. Moreover, because it establishes a strong lateral connection between the talus and the calcaneus, this ligament also prevents excessive inversion [4]. In contrast, the ITCL hinders excessive eversion and pronation movements at the subtalar joint. The ITCL is formed partially by the fusion of the capsules of the anatomical talocalcaneonavicular and subtalar joints [8]. Variations of the ITCL were noted (band type, fanlike and multiple types) in different cadavers, which were confirmed in our study [8]. While the fiber orientation reported by Kelilian was superomedial to inferolateral at an oblique angle [8], as observed in our study for most cadavers, we found that the ITCL fibers lay in a superolateral to the inferomedial direction in a few specimens. Variations in fiber orientation of this ligament should be taken into account for screw placement during subtalar fusion or subtalar ligament reconstruction.

The clinical significance of the morphometrics and fiber orientation of the CL and ITCL becomes crucial when subtalar instability is assessed and treated. Since most subtalar ligament injuries are believed to occur concomitantly with injuries to the lateral ankle ligaments, these two ligaments should also be assessed when inversion ankle sprains are treated [4]. One method for assessing the subtalar ligaments is by stress radiography, in which the degree of talar tilt is measured. Another method is the medial subtalar glide test, which measures the extent to which the calcaneus translates medially on the talus in the axial plane [1]. Subtalar instability results from loosening or tears of the CL and ITCL, which are often overlooked during diagnosis of lateral ankle sprains. In addition, owing to pain and swelling situated in the sinus tarsi as a result of the rupture of the ITCL, it has occasionally been diagnosed erroneously as sinus tarsi syndrome [4]. Currently, the most desirable surgical treatment for painful subtalar instability is subtalar fusion, also known as arthrodesis [9]. However, successful cases of reconstructive repair of the ITCL under subtalar arthroscopy have been reported [2].

Conservative treatment such as the use of a brace should be considered first with surgical treatment and should be reserved for extremely painful cases as incomplete rupture of the ITCL [2]. The use of a gracilis tendon autograft [4] and a piece of Achilles tendon are both described as methods to replace the ruptured ITCL. After the bony tunnels were created in both the talus and the calcaneus, the new ligament was passed through the tunnels and in the same direction (superomedial to inferolateral) as the ITCL before the injury. However, no comments were made regarding the variations in fiber orientation of the ITCL. While it is true that the most common fiber orientation is superomedial to inferolateral, variations such as seen in our study should be taken into consideration for better outcomes in terms of talocalcaneal stabilization. In addition to demonstrating the site of rupture of the ITCL, a magnetic resonance imaging (MRI) scan also yields important information regarding the fiber orientation of this ligament for reconstruction purposes.

The statistical analysis demonstrated a strong correlation between the talar neck width and CL thickness, which is of clinical significance for roentgenometrics. One can deduce the approximate thickness of the CL by measuring the width of the talar neck on the plain radiograph with the dorsoplantar view. Further, the CL is much thicker posteriorly than anteriorly. The other variables such as talar neck height and CL thickness as well as foot length and talar neck height were only moderately correlated. Further studies are needed to confirm the strong correlation between talar neck width and CL thickness by reproducing this study with a larger sample.

## Conclusions

Our findings can improve understanding of the relationship between the cervical ligament (CL) and interosseous talocalcaeal ligament (ITCL), which are the two ligaments frequently injured within the tarsal sinus. In order to prevent chronic subtalar instability, clinicians should consider repairing or stabilizing the appropriate subtalar ligaments depending on the severity of inversion or eversion injury. Thus, the morphometric data from our study can assist podiatric surgeons in reconstructing these ligaments accurately to stabilize the subtalar joint better. Future studies should focus on reconstruction of the ITCL on the basis of variations in fiber orientation and subsequent assessment of functional stability.
